# Mycotoxins in Cheese: Assessing Risks, Fungal Contaminants, and Control Strategies for Food Safety

**DOI:** 10.3390/foods14030351

**Published:** 2025-01-22

**Authors:** Camila Aranda, Rodrigo Rodriguez, Martín A. Fernández-Baldo, Paola Durán

**Affiliations:** 1 Programa de Doctorado en Ciencias de Recursos Naturales, Universidad de La Frontera, Temuco 4811230, Chile; s.aranda01@ufromail.cl; 2 Biocontrol Research Laboratory, Universidad de La Frontera, Temuco 4811230, Chile; r.rodriguez09@ufromail.cl; 3 AgroDNA SpA, Pedro de Valdivia 0380, Temuco 4811230, Chile; 4 Instituto de Química San Luis (INQUISAL), Departamento de Química, Universidad Nacional de San Luis, CONICET, Ejército de los Andes 950, San Luis D5700BWS, Argentina; mbaldo@unsl.edu.ar; 5 Facultad de Ciencias Agropecuarias y Medio Ambiente, Universidad de La Frontera, Temuco 4811230, Chile

**Keywords:** filamentous fungi, mycotoxin contamination, food security, cheese production

## Abstract

According to the scientific information reviewed, cheese is highly susceptible to contamination by mycotoxin-producing fungi, primarily species from the genera *Aspergillus* (*A. niger*, *A. flavus*) and Penicillium (*P. commune*, *P. solitum*, *P. palitans*, and *P. crustosum*). Studies on various types of cheese made from cow’s milk report an average concentration of Aflatoxin M1 (AFM_1_) at 13,000 ng kg^−1^, which is alarming since the regulatory limits for AFM_1_ in cheese range from 250 to 500 ng kg^−1^. For instance, limits set by Codex Alimentarius, the European Commission (EC), Turkey, and Iran are 250 ng kg^−1^. In the Netherlands, the limit is 200 ng kg^−1^, and in Italy, it is 450 ng kg^−1^. However, the concentration of mycotoxins frequently exceeds these regulatory limits, including critical mycotoxins such as ochratoxin A, citrinin, and cyclopiazonic acid, which pose significant global health concerns. Therefore, this study aims to review the mycobiota responsible for producing key mycotoxins in cheese and to assess the influence of physicochemical factors on fungal growth and mycotoxin production. By incorporating control strategies such as hygiene practices, pasteurization, and the use of preservatives, this study seeks to improve methodologies in the cheese production chain and mitigate contamination by fungi and mycotoxins.

## 1. Introduction

The global dairy trade is projected to expand over the next decade, reaching 14.2 Mt by 2031, which is 15% higher than the base period, with cheese trade expected to grow at a rate of 1.6% per year [1]. Europe is the largest consumer of cheese, accounting for more than 60% of the global consumption. Other significant cheese-consuming regions include North America, Asia–Pacific, and Latin America. Cheese production involves a series of biochemical processes, using cow, goat, or sheep milk—or a mixture of these—as the raw material, along with the addition of sodium chloride (NaCl) and coagulant additives. Furthermore, a dynamic microbial community composed of filamentous fungi, yeasts, and bacteria plays a crucial role in the cheese-making process [2,3]. Nowadays, it is estimated that more than 1000 types of cheese are available in the global market, varying in flavor, aroma, texture, color, appearance, yield, and nutritional composition [4,5].

Cheese is a substrate rich in proteins, fats, mineral salts, and vitamins, making it highly susceptible to microbial growth. Microbial succession plays a vital role in developing the distinctive biochemical and organoleptic traits in cheese, particularly during the ripening stage [6,7]. Although diverse, the microbiota associated with cheese can be divided into starter lactic acid bacteria (LAB) and, in secondary microbiota, “fungal species that are not part of the primary or dominant microbiota”, which can include filamentous fungi and yeast [8,9]. Among the secondary microbiota, some filamentous fungal strains, such as *Penicillium camemberti* and *Penicillium roqueforti*, may be deliberately introduced during cheese-making to enhance and improve the final product. However, filamentous fungi are particularly significant due to their potential to produce mycotoxins. The uncontrolled growth of these fungi can lead to undesirable effects in cheese. Contamination with filamentous fungi can occur at various stages of cheese production, including in the milking parlor, storage, ripening, packaging, and transportation [10]. Thus, intentionally added strains increase the risk of uncontrolled fungal growth and mycotoxin production, which is especially critical in the production of artisanal cheeses, as the potential for mycotoxin production in the final product has not been thoroughly evaluated [11].

Mycotoxins are low-molecular-weight secondary metabolites produced by filamentous fungi with adverse effects on human and animal health [12,13,14,15]. Spoilage fungi isolated from cheese have been reported as aflatoxins (AFs) and ochratoxin A (OTA) producers, which are among the most dangerous known mycotoxins [16,17,18,19,20,21,22]. Although less toxic, mycotoxins like citrinin (CIT), cyclopiazonic acid (CPA), penitrem A (PA), roquefortine-C (ROQ-C), and mycophenolic acid (MPA) have also been reported in different cheese types [23,24,25,26,27,28]. Despite *Penicillium camemberti* and *Penicillium roqueforti* having the Generally Recognized as Safe (GRAS) status, they have been described as producing a detectable amount of CPA, *Peniciliium roqutforti* toxin (PR toxin), and ROQ-C [12,16,29]. Although these mycotoxins are considered to be of low toxicity, the combined intake of different mycotoxins may lead to a synergistic or at least additive effect [30].

From an ecophysiological point of view, cheese is a highly complex food substrate, with intense microbial interaction. Until now, there has been a lack of information about how heterogeneous physicochemical composition throughout cheese production (e.g., pH, NaCl, moisture, water activity (a_w_), O_2_) can affect the growth of spoilage fungi and mycotoxin production. Currently, analytical methodologies for the determination of mycotoxins in food samples are High-Performance Liquid Chromatography (HPLC), gas chromatography, mass spectrometry, and commercial enzyme-linked immunosorbent assay [31]. These methods are accurate and sensitive but require qualified personnel and high costs, and the sample pre-treatment is time-consuming. In recent years, important biosensors have been developed for mycotoxin determination in different matrices of food samples. These analytical devices consist of a biological element for recognizing the analyte and a transducer, which translates the biorecognition event into a signal proportional to the analyte concentration [32]. However, very few biosensors have been reported to date for the detection of mycotoxins in cheese samples, which could open an interesting door for research. Biosensors can represent interesting alternatives to traditional methods and are characterized by the specificity given by the biorecognition element (specific monoclonal antibodies, DNA, or RNA), sensitivity, rapid response, portability, multimodal detection, and the possibility of in situ application for the determination of different mycotoxins.

To contribute to a better understanding of the occurrence and role of potential mycotoxigenic fungi and mycotoxin production in cheese, the aim of the present study is to review the main mycotoxins reported in cheese and evaluate the influence of physicochemical factors on the growth of potential mycotoxigenic fungi and their production of mycotoxins. Additionally, the best practices for controlling fungal spoilage and mycotoxin production in cheese will be reviewed and discussed. Despite extensive studies on mycotoxin contamination in foods, few reviews have comprehensively addressed mycotoxin control and detection strategies specific to cheese and the mycotoxins produced by filamentous fungi.

## 2. The History of Cheese-Making: Microbial Succession and Its Role in Flavor and Safety

Cheese is a dairy product obtained after milk fermentation. Ancient civilizations converted milk into cheese apparently as a strategy to facilitate storage and transportation and to diversify the human diet [14,19]. Considering the long history of cheese-making, different varieties of cheese and manufacturing methods have been developed throughout human civilization history. Overall, cheese-making can be summarized into three main steps. In the first one, the milk source is selected as cattle, water buffalo, sheep, or goat. Cheese can be produced from a single milk type or a blend of milk types. Single or blended milk is then coagulated to be transformed into solid curds and liquid whey. This process is carried out by acidification and proteolysis. Acidification occurs when LAB ferments lactose to produce lactic acid. The ecology and function of these primary microbial starters in different cheese types have previously been reported [10,33,34]. In the second step, curds containing casein and milk fat are separated from whey. Depending on the cheese type, the curds can be heated, salted, and pressed, acquiring a different shape and texture. The third and last cheese manufacturing step is ripening. The main cheese transformations in organoleptic properties occur in this stage, where cheese acquires specific characteristics of flavor, aromas, and texture [3,33,35]. Slight modifications throughout these stages, such as rheological changes, can produce cheese with different consistency such as fresh, soft, or hard cheese.

In addition to the coagulation stage, pH value, NaCl, and O_2_ concentrations are determinants to set up the cheese microbiota [20,24,25]. Microbial succession has a key role throughout the cheese-making steps and includes starter LAB. Non-starter LAB (NSLAB) refers to naturally occurring LAB that are not intentionally added as part of the starter culture (e.g., *Pediococcus*, *Enterococcus*, and *Leuconostoc*); propionic acid bacteria, which use the propionic acid fermentation pathway, converting lactate (produced by lactic acid bacteria) into propionic acid, acetic acid, and carbon dioxide; yeast; and filamentous fungi [36]. Previous studies have shown the diversity of bacteria and yeast in cheese [2,6,10]. The role and interaction of indigenous and spoilage mycobiota and the introduced fungal cultures throughout cheese manufacturing are important steps within microbial succession, with an impact on the organoleptic properties and food safety of cheese [37].

## 3. The Role of *Penicillium* and Other Filamentous Fungi in Cheese-Making: From Flavor Development to Spoilage Risks

Filamentous fungi found in cheese can be introduced during cheese-making through the use of commercial ripening cultures or may be adventitious, originating from the environment or raw materials such as milk. *Penicillium* species are commonly reported as the dominant spoilage fungi in cheese, mainly *P. commune*, *P. solitum*, *P. palitans*, and *P*. *crustosum* species. The psychrotolerant and xerotolerant characteristics of some *Penicillium* species confer to them a competitive advantage over other microorganisms, especially during cheese ripening and subsequent storage and retailing [38]. *Penicillium camemberti* and *P. roqueforti* have traditionally been used as secondary starters in surface fungal-ripened and blue-veined cheeses, respectively [25,39]. *Penicillium roqueforti* is used as a secondary starter culture for the ripening of blue cheese such as Roquefort, Gorgonzola, Stilton, Danablu, Cabrales, Bleu d’Auvergne ort, Blue de Bresse, and Blue Stilton. The proteolytic and lipolytic activity of *P. roqueforti* influences the aroma and flavor of blue cheese [39,40,41]. *Penicillium roqueforti* grows throughout the internal fissures between the incompletely fused curd particles, corresponding to approximately 80% of the mycobiota of blue cheese. It is well adapted to cheesemaking environments, as it is able to grow in low levels of oxygen (range of 0.3–21%) [17,42]. On the other hand, *P. camemberti* is used in cheese-making because of its high proteolytic and lipolytic activities, which are important contributors to the development of the typical taste of soft cheeses such as Brie, Camembert, Coulommiers, and Neufchâtel [22,28].

The mycobiota of the cheese-making environment is essential to confer the unique flavor of artisanal cheese [38,43]. Previous studies have reported that horizontal transfers of large genomic regions (HTRs) play an important role in fungal adaptation to cheese-making environments. These regions are of particular relevance since they carry crucial metabolic genes [4,44,45]. According to Anelli et al. [38], *Penicillium gravinicasei* showed rapid growth in a medium containing 5% NaCl, indicating the adaptation of this fungal species to the cheese salting process in cheese ripening. Its halotolerance, enzymatic resilience, and ability to thrive in low-water-activity environments make it a key contributor to high-quality and flavorful cheeses [38]. Thus, an uncontrolled outgrowth of spoilage fungi in cheese may produce off-flavor, surface discoloration, and mycotoxin contamination, which can affect cheese quality and its commercialization [45]. Spoilage fungi can be found during the early stages of cheese-making, such as in the shed and milking parlor environments. Moreover, fungal spores, which are found in handled and stored cheese, are easily dispersed into dairy plant air [39,40].

Lund et al. [46] analyzed the mycobiota of hard, semi-hard, and soft cheeses produced in African (e.g., South Africa), American (Costa Rica and USA), Asian (Japan), European (e.g., Azores, Belgium, Czechoslovakia, Czech Republic, Denmark, France, Germany, Greece, Italy, Malta, Netherlands, Norway, Spain, Switzerland, and United Kingdom), and Oceanian countries (Australia and New Zealand). According to the authors, *Penicillium commune*, *P. palitans*, *P. nalgiovense*, and *P. verrucosum* were the most frequent contaminant fungi in cheese samples.

Montagna et al. [47] analyzed the mycobiota of goat, sheep, and goat/sheep blend cheeses produced in Southern Italy through handmade or industrial manufacture. One hundred and twenty-two cheese samples were analyzed. From these, 66 samples were obtained from industrial manufacture, while 56 were obtained from handmade manufacture. Spoilage fungi were found in 54 samples (44.3%), predominantly in industrialized samples (59.1%) rather than in artisanal samples (26.8%). The short timeframe between the maturation, commercialization, and consumption of artisanal cheeses may have contributed to avoiding the growth of filamentous fungi [47]. According to the authors, *Penicillium* was the most frequently fungal genera in cheese samples (72.9%). Of these, the species *P. brevicompactum* (11, 46%), *P. commune* (15%), and *P. solitum* (11.46%) were the most frequent ones. Species of *Geotrichum* (7.3%), *Aspergillus* (4.2%), and *Mucor* (4.2%) were also isolated.

Finne et al. [48] reported that *Penicillium* species were isolated from c.a. 89% of the semi-hard cheeses Norvegia and Jarlsberg produced in Norway. The most frequently occurring species was *P. roqueforti*. Also, *P. commune*, *P. palitans*, and *P. solitum* corresponded to c.a. 70% of the total fungal strains isolated from both cheeses. *Penicillium commune* appears to be well adapted to cheese. It is the wild-type species of the domesticated form of *P. camemberti* [48]. In addition to *Penicillium*, the genera *Aspergillus*, *Alternaria*, *Cladosporium*, *Fusarium*, *Geotrichum*, *Mucor*, and *Trichoderma* have also been reported as cheese contaminants (Table 1). Moreover, *Aspergillus niger* and *Aspergillus flavus* are also reported as fungal spoilage in cheese [42,49,50].

### 3.1. Unveiling the Hidden Threats: Fungal Mycotoxins in Cheese and Cutting-Edge Biosensors for Detection

Filamentous fungi can produce several secondary metabolites. Some of them have potential biological and pharmacological activities, while others, classified as mycotoxins, present adverse effects on human and animal health [25,28,41,58,59]. Some mycotoxins are carcinogenic or genotoxic or may affect the kidneys, liver, and other organs and the immune system [17,60,61]. Previous studies have described the presence of fungal secondary metabolites in cheese, including several mycotoxins [36,62].

It has been suggested that the conditions of the cheese maturation room (e.g., temperature, water activity) may favor fungal growth and mycotoxin production [20,28,57]. However, visible fungal colony development in cheese and mycotoxin concentration are not always correlated [60,61,62,63]. Among mycotoxins in cheese, Afs (Aflatoxins), CIT (Citrinin), OTA (Ochratoxin A), PA or PAT (Patulin), ROQ-C (Roquefortine C), and STC (sterigmatocystin) are common contaminants in cheese [2,35,36,62,63]. Table 2 summarizes the main mycotoxins reported in different cheese types.

In recent years, the same relevant biosensors have been developed for mycotoxin determination in cheese samples. Hossain et al. (2019) reported an immunosensor for detecting CPA combining an indirect competitive immunoassay and signal amplification based on a secondary antibody conjugated with gold nanoparticles [75]. Furthermore, Varlamova et al. (2019) proposed an amperometric biosensor based on a screen-printed platinum electrode modified by carbon nanotubes, graphene oxide, and gold nanoparticles in chitosan and immobilized the enzyme tyrosinase applied to AFM_1_ determination [76].

### 3.2. Aflatoxins: Carcinogenic Contaminants in Cheese Production

Aflatoxins are mainly produced by strains of *Aspergillus flavus* and *A. parasiticus* and rarely by *A. nominus*, *A. tamarii*, and *A. pseudotamarii*. Aflatoxigenic fungal strains can contaminate crops such as maize that are used as cattle feed [77,78] or as an ingredient for balanced animal feed [49,60]. The best conditions of temperature and moisture for the growth of these fungal species are between 24 °C and 35 °C and above 7%, respectively [17,26]. There are more than 300 known mycotoxins. Among them, AFB1 and AFM_1_ are included in the most toxic ones and are classified by the International Agency for Research on Cancer (IARC) as Group 1 of human carcinogens [17]. AFM_1_ is a hydroxylated metabolite of AFB1. AFM_1_ results of AFB1-contaminated feed ingestion by ruminant animals. A fraction of the ingested AFB1 is degraded in the rumen, resulting in the formation of aflatoxicol. The remaining fraction is absorbed in the digestive tract by passive diffusion and is hydroxylated in the liver to AFM_1_, which is left in the milk, urine, and other body fluids of animals intoxicated with AFB1 [2].

Cheese produced from AFM_1_-contaminated milk is a major concern because the final product will be indirectly contaminated with this mycotoxin. The conversion rate of AFB_1_ from feed to AFM_1_ in milk (residual) depends on several factors and generally ranges from 2% to 6%. Consequently, AFM_1_ levels in milk can vary depending on geographic location, level of economic development, and climatic conditions [41,54,79,80,81]. AFM_1_ is relatively resistant to heat treatments, such as milk pasteurization, and chemical processes used during cheese production, including pH changes [42,56]. In addition, it has been demonstrated that AFM_1_ binds to milk proteins, resulting in its uneven distribution between whey and curd, with the highest concentration found in the curd. If AFM_1_-contaminated milk is used to produce dairy products, AFM_1_ levels in the final product can be up to three times higher than in the milk [35,61,82,83,84,85,86]. Cheese contamination with AFM_1_ has frequently been reported as a serious food safety issue in developing countries [61,86]. Currently, mycotoxin regulations for cheese primarily focus on aflatoxin M1 (AFM_1_). Several countries and economies worldwide have established maximum tolerable limits (MTLs) for AFM_1_ in milk and dairy products. According to the European Commission Regulation, the MTL for AFM_1_ in cheese is 0.05 μg kg^−1^. In both Australia and Honduras, the MTL is 0.25 μg kg^−1^, while in the United States (USA), it is set at 250 ng kg^−1^, and in Italy, it is 450 ng kg^−1^. On the other hand, in Brazil, regulations focus on total aflatoxins (AFs) in cheese, with the maximum tolerable limit (MTL) set at 2.5 μg kg^−1^ [8,87].

Anfossi et al. [88] conducted a one-year survey measuring AFM_1_ contamination in cheese purchased from Italian markets. Over 100 samples, representing a wide variety of cheese types, origins, cheese-making, and maturation processes, were analyzed. More than 83% of the samples showed detectable levels of AFM_1_ above 25 ng kg^−1^, with contamination ranging between 50 and 150 ng kg^−1^. AFM_1_ concentration was correlated with four factors presumed to influence contamination levels: manufacturing, production season, milking, and maturation. The authors noted that milking and manufacturing processes significantly affect AFM_1_ concentration, as cheese made from cows’ milk and artisanal production methods showed higher contamination levels than cheese produced with milk from other animals, such as sheep and goats, in industrial contexts. This class of mycotoxins is known to cause severe liver damage and promote tumor formation and is associated with immunosuppressive, mutagenic, teratogenic, and carcinogenic effects [17,89,90,91,92].

### 3.3. Ochratoxin A: A Salt-Tolerant Mycotoxin in Cheese Maturation

Ochratoxin A (OTA) OTA has carcinogenic, nephrotoxic, immunotoxic, teratogenic, neurotoxic, and genotoxic effects [65]. It is also recognized for causing severe pathological responses in humans and animals [22,56,92,93,94,95]. Due to growing concerns, OTA has been classified by the IARC as a Group 2 human carcinogen. OTA is a chlorinated polyketide mycotoxin that contains the amino acid phenylalanine in its molecule [65]. Its constant biosynthesis in Penicillia ensures the permanent excretion of chlorine from the cell [96]. OTA production is directly proportional to the fungal growth rate under high NaCl conditions. As a result, high OTA-producing fungal strains are better adapted to compete in salt-rich habitats compared to strains that produce lower amounts of OTA [13,39,97,98]. Given this, it can be presumed that cheese, due to its NaCl content (among other abiotic factors), provides a favorable environment for OTA production. Therefore, the effect of NaCl concentration on OTA production in cheese must be considered alongside other physicochemical factors such as temperature, water activity (a_w_), and pH. Thus, NaCl content can have a different impact on the production of other mycotoxins. Finoli et al. [51] evaluated the impact of abiotic factors, including NaCl content, on the production of ROQ-C precursors, ROQ-C, and MPA by 96 strains of *P. roqueforti*. They observed a significant decrease in the production of ROQ-C and MPA in the presence of 8% NaCl, indicating that NaCl negatively impacts the production of these mycotoxins.

About 20 fungal species are known potential OTA producers. The most significant among them are *A. ochraceus*, *A. steynii*, and *A. westerdijkiae* (genus *Aspergillus* section *Circumdati*); *A. carbonarius* and *A. niger* (genus *Aspergillus* section *Nigri*); and *Penicillium nordicum* and *P. verrucosum* (genus *Penicillium* section *Viridicata*) [95]. OTA has been detected in various cheese types [99,100]. In blue cheese, OTA levels range from 0.02 to 2.5 μg kg^−1^ [91]. In traditional semi-hard cheese, concentrations vary from 1 to 262 μg kg^−1^ in the bark and from 18 to 146 μg kg^−1^ on the cheese surface. In mature cheese, OTA levels range from 0.2 to 317 μg kg^−1^ [54]. Recently, Pietri et al. [100] reported that the surface of 16 hard grana cheese rinds was contaminated with OTA at concentrations ranging from 3 to 370 μg kg^−1^. Additionally, Leggieri et al. [25] reported that OTA concentrations in grana cheese samples from various stores in Italy varied between 1 and 1432 μg kg^−1^, with an average concentration of 183 μg kg^−1^. Surprisingly, the fungal species isolated from these cheese samples have not been described as OTA producers. This suggests that some isolated strains may be from OTA producers, or the mycotoxin could be present in the raw milk used in Grana cheese production. Moreover, OTA concentration was higher compared to a similar study by Biancardi et al. [64], in which hard cheese samples from small businesses in northern Italy had OTA concentrations ranging from 1.62 to 54.07 μg kg^−1^.

To provide useful information on the origin of OTA contamination and to develop effective prevention methodologies, it is crucial to closely monitor the various stages of traditional cheese production processes [24,53]. Thus, the identification of emerging OTA-producing fungal strains requires a combination of advanced molecular, metabolomic, and genomic approaches (e.g., emerging technologies like biosensors, nanotechnology, and AI), while studies on fungal evolution highlight the impact of environmental and human factors on mycotoxin production.

### 3.4. Other Relevant Mycotoxins: Emerging Risks in Cheese Safety

Other mycotoxins with less toxicity have been reported in cheese [14]. Izzo et al. [73] demonstrated that, out of all detected metabolites in traditional Slovak cheeses, enniatin B represents the most frequently detected mycotoxin (0.06–0.71 μg kg^−1^) in the analyzed samples. However, enniatins do not have globally established maximum permissible levels in food by regulatory bodies like the European Food Safety Authority (EFSA) or the US Food and Drug Administration (FDA). Finoli et al. [51] evaluated the presence of ROQ-C and PR toxin in 86 Gorgonzola blue cheese samples produced in Italy. According to the authors, 97.7% and 37.2% of cheese samples contained quantifiable levels of ROQ-C and MPA, respectively, with mean levels of 848 ± 1670 μg kg^−1^ for ROQ-C and 841 ± 1271 μg kg^−1^ for MPA. The authors point out that these results could be associated with the long maturation periods to which cheese is submitted, from 8 to 20 weeks, and with the toxigenic potential of the maturing fungal cultures used. Variations in blue vein cheese compositions or cheese-making processes could also lead to the expression of mycotoxins in the cheese matrix by potentially toxigenic fungal strains.

Bailly et al. [53] analyzed the capability of *Penicillium citrinum* and *P. expansum* strains to produce CIT on goat cheese. The authors showed that both fungal strains were able to produce CIT on fresh goat cheese kept at 20 °C for 7 days. CIT production on fresh goat cheese was higher than that produced on YES medium. These data suggest that fresh goat cheese may be a good substrate for mycotoxin production by *Penicillium* species. Furthermore, in all tested cases, more than 50% of the initial CIT content was still present after 8 days of storage. Regarding CIT diffusion, 96% of the produced mycotoxin was contained in the upper 0.5 cm cheese layer. Despite being considered to be of low toxicity, CIT stability on cheese is a worrying factor since the interaction between different types of mycotoxins may lead to a synergistic or at least additive effect [59]. However, currently, there is no legislation to regulate the combined contamination of mycotoxin in cheese. Coton [22] demonstrated that, in semi-hard Comté cheese, by quantifying the accumulation and migration of citrinin (CIT) and ochratoxin A (OTA) after artificial inoculation with a producing *P. verrucosum* strain at 8 °C, the production of CIT and OTA started after 14 days and 28 days of incubation, respectively; whereas at 20 °C, both mycotoxins were produced from Day 7 onwards. At 20 °C, the maximum concentration of CIT, approximately 50,000 ng/g, was 20 times higher than at 8 °C. The maximum concentrations were obtained at the top of the cheese, but depending on the incubation time, mycotoxins were detected up to 1.6 cm deep.

Decontardi et al. [28] collected eighteen samples of Grana cheese and evaluated cultivable mycobiota and mycotoxin production. CIT was detected in four cheese samples ranging from 42 to 100 μg kg^−1^. Cakmakci et al. [55] evaluated 140 *Penicillium roqueforti* strains isolated from 41 samples of fungi-ripened civil cheese (traditional cheese using skimmed milk and the addition of whey in its manufacture) produced in Erzurum Province in Eastern Turkey. All strains were capable of producing ROQ-C, penicillic acid, MPA, and PAT. The amounts of these mycotoxins ranged from 0.4 to 47.0; 0.2 to 43.6; 0.1 to 23.1; and 0.1 to 2.3 mg kg^−1^, respectively. Thus, mycotoxin interactions often have synergistic or additive effects, increasing health risks. These interactions are significant in contexts like dietary exposure, livestock feed, and food safety. Current regulatory frameworks focus on single toxins, highlighting the need for integrated risk assessments and advanced multi-toxin detection technologies.

### 3.5. Impact of Physicochemical Conditions on Mycotoxin Formation in Cheese

**Figure 1:**
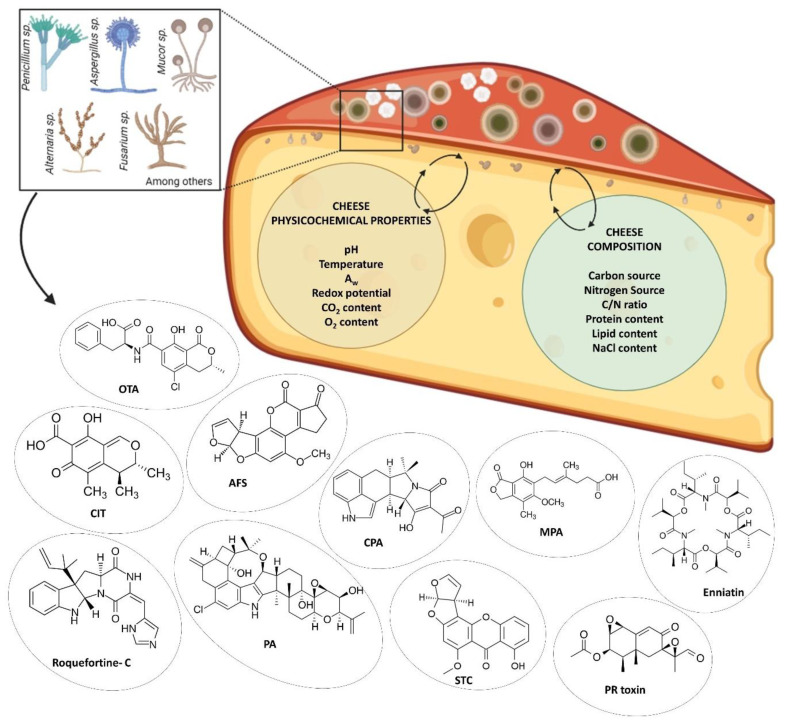
The key physicochemical parameters that affect fungal growth and mycotoxin production in cheese. These include temperature, water activity (a_w_), pH, NaCl content, moisture, carbon and nitrogen sources, C/N ratio, and redox potential (E°). The interaction of these factors determines fungal spore germination, colony growth, and toxin synthesis, highlighting their critical roles in cheese contamination dynamics.

The mechanism of mycotoxin production in cheese is not yet clear, especially considering that mycotoxin production can occur as a fungus response to different ecological scenarios. Mycotoxin production and secretion can be used as chemical signals for communication and as a competitive strategy to defend the habitat against microorganisms of the same trophic niche. It is important to note that associated with the mycobiota assemblage, substrate physicochemical factors are determinants of mycotoxin production [23,71,72]. Therefore, considering cheese as a substrate, the main physicochemical factors determining fungal growth include temperature, moisture content, a_w_, pH, the cheese own chemical composition, carbon and nitrogen sources, C/N ratio, NaCl content, and redox potential (E°). These factors would play a fundamental role in fungal spore germination, fungal growth, and mycotoxin production (Figure 1) [56,57,101,102].

### 3.6. Temperature, Water Activity, and pH: Key Drivers of Mycotoxin Production in Cheese

Environmental temperature is an essential physicochemical factor for mycotoxigenic fungal growth and mycotoxin production in cheese. Mycotoxin production is inversely related to temperature with little or no production at temperatures < 12 °C, intermediate production at 12 °C, and optimal production from 20 to 25 °C [87]. It is thus not surprising that cheese storage at temperatures ranging from 5 to 7 °C is vital to prevent mycotoxigenic fungi growth and cheese contamination with mycotoxin [103]. At these temperatures, *Penicillium* species are among the most commonly encountered food spoilage fungi capable of growing in diverse conditions, but other fungal species may also contribute to spoilage depending on the food matrix and environmental factors [13].

One of the determining factors for the production of mycotoxins is temperature [23]. Abd Alla et al. [104] reported that the temperature value for Ras cheese ripening affected the production of the STC. According to the authors, a temperature of 6 °C used for ripening cheese led to a weak (125 μg kg^−1^) production of STC, while at 20 °C, the STC concentration was higher (250 μg kg^−1^). By integrating these findings, it becomes evident that temperature is a critical factor in managing fungal contamination during cheese ripening, though strain-specific behaviors warrant further investigation to develop tailored control strategies.

The sporulation of filamentous fungi has been previously shown to be dependent on thermodynamic factors such as water availability because they are critical factors affecting the growth and metabolism of fungi [69,105,106]. Parra et al. [107] evaluated that optimal growth of three strains of *A. niger* examined to be in the range 0.97–0.95 a_w_.

Previous studies have shown that the observed a_w_ × temperature conditions for fungal germination are broader than those observed for mycotoxigenic fungal growth and mycotoxin production [13,101]. Predictive contour maps for mycotoxin production risk are now available for a wide range of mycotoxigenic fungal species growing in environmental conditions with different physicochemical factors. Thus, the optimum a_w_ and temperature growth for *Aspergillus flavus* has been found to be from 0.98 to 0.99 and from 30 to 35 °C, respectively. However, for this fungal species, the best conditions for AFB1 production were found to be at a_w_ from 0.95 to 0.99 with temperatures ranging from 25 to 35 °C [13,98]. For *Fusarium verticillioides*, the optimal a_w_ and temperature growth conditions were at 0.99 and 25 °C, respectively, as well as at 0.98 and 30 °C, respectively. For this fungal species, AFB1 and AFB2 biosynthesis was observed to be optimal at a_w_ 0.98 and 20 °C, and at 0.99 and 25 °C, respectively [97].

Water activity and temperature interaction over time are the main factors to produce fungal spores, especially in the cheese matrix [9,54,57]. Fungal sporulation, growth, and mycotoxin production find an adequate regimen during cheese ripening. Therefore, a clear understanding of fungal sporulation as the primary source of inoculum for cheese colonization is crucial [54]. Pascual et al. [72] studied in vitro the effect of temperature, pH, water potential, and sources of nitrogen and carbon on the biocontrol agent *Penicillium oxalicum*. For this, the authors used as parameters fungal sporulation, spore germination, germ tube length, and colony growth rate. Based on the results, the authors observed different fungal sensitivities to environmental factors and nutrients. Sugars such as xylose, mannose, and fructose led to the highest fungal growth rates, while mannose induced strong sporulation. Peptone and free amino acids gave both the highest fungal growth rates and high levels of sporulation. The authors concluded that the fungus has a xerotolerant character. Moreover, the isolate of *P. oxalicum* could be an effective biocontrol fungus because it has the potential to grow well in a wide range of environmental conditions. Similarly, Cheeseman et al. [39] and Decontardi et al. [57] reported uncontrolled fungal growth on the surface of different cheese types during cheese ripening and aging. According to the authors of both studies, the cheese ripening and aging processes can lead to fungal growth and mycotoxin contamination. In studies conducted by Erdogan and Sert [9], in Tulum cheese inoculated with *P. roquefort* matured at 5 °C and 12 °C for four months, ROQ-C was detected, the concentration of which increased throughout the maturation of the cheese. The amount of ROQ-C found in the matured cheese at 5 and 12 °C for the first month was 2.1 to 2.4 mg kg^−1^, and at four months, it was 2.1 to 3.8 mg kg^−1^. In addition, the authors showed that the occurrence of ROQ-C increased in parallel with a higher maturation temperature (12 °C). Leggieri et al. [57] modeled in vitro the effect of temperature and a_w_ conditions on the growth of *Aspergillus versicolor*, *Penicillium camemberti*, *P. citrinum*, *P. crustosum*, *P. nalgiovense*, *P. nordicum*, *P. roqueforti*, and *P. verrucosum* and mycotoxin production on Italian Grana cheese. Fungal strains grew under temperature regimes from 0 to 40 °C and a_w_ values from 0.78 to 0.99. According to the authors, all the fungi were highly susceptible to a_w_, which was 0.99 for the fungal growth-optimal a_w_ value for almost all species, except the more xerophilic *A. versicolor*, which grew best with a_w_ = 0.96. In addition, the highest relative growth occurred around 25 °C, and the optimum a_w_ was 0.96 for *P. crustosum*, *P. nordicum*, and *P. verrucosum*. Also, for these species, highest mycotoxin production occurred between 15 and 25 °C and with a_w_ values from 0.96 to 0.99. According to the authors, reducing the temperature and a_w_ factors in ripening and cheese crust will make conditions more limiting for the development of mycotoxigenic fungi.

The regulatory pathways involved in mycotoxin biosynthesis by various fungal species including *Penicillium* spp. can be influenced by pH, but little information is available about the effects of pH on CPA production by cheese-related fungi [21,96]. Little information is also available on the effect of pH, a_w_, and temperature on CPA production by cheese-related fungi. Casquete et al. [23] evaluated the influence of pH, a_w_, and temperature in the phases of retardation, fungal growth, and CPA production by *Penicillium commune* (CBS311 and CBS341) and *P. camemberti* CBS273 in a cheese-based medium. According to the results, the behaviors of *Penicillium* strains were influenced by pH, a_w_, and temperature. *Penicillium commune* CBS311 and CBS341 showed the highest specific growth rate (*p* < 0.05) at pH 5.0, a_w_ 0.99, and 25 °C; while *P. camemberti* CBS273 showed a maximum growth rate at pH 5.0 and 5.5, a_w_ 0.99, and 25 °C. Overall, although a_w_ and temperature were the most limiting factors, low a_w_ and temperature, or high pH, increased latency times and had the greatest impact on the growth rate and diameter of the colony. In addition, for *P. commune* CBS311 and CBS341, the maximum CPA production on cheese-based medium was at pH 5.0, a_w_ 0.99, and 25 °C, respectively. In contrast, *P. camemberti* CBS273 displayed a maximum growth rate under four different conditions, specifically, pH 5.0 and 5.5, a_w_ 0.99, 25 °C, and pH 5.0, a_w_ 0.95, at 20 and 25 °C [23].

### 3.7. Preventing Mycotoxin Contamination: The Role of Hygiene and Control Strategies

As discussed above, the production of different cheese types may be under contamination risk due to highly toxic mycotoxins, such as OTA, AFB1, AFM_1_, and others [25,28,41,108]. In this way, it is essential to carry out preventive actions to control mycotoxin contamination in cheese (Table 3). Therefore, these actions should strictly consider (I) the implementation of good hygienic practices, which includes (II) pasteurization, (III) the application of chemical or (IV) natural preservatives, and (V) the use of biological control. These preventive actions are essential to minimize the growth of mycotoxigenic fungi and mycotoxin contamination in the cheese production chain [22,109,110,111].

The strict application of good hygiene practices before, during, and after milking is essential. In addition, special care must be taken in the application of a sanitation schedule to the cheese processing plant, including the maturation room, equipment, and utensils used in the manufacture of cheese. Also, in order to reduce the occurrence of putative mycotoxigenic fungi, which occur naturally in the air environment of facilities, the proper hygiene of the personnel is essential [33,45,112,113].

### 3.8. Pasteurization: A Critical Step to Minimize Mycotoxin Risks in Cheese

Pasteurization is a critical step in the cheese-making process to minimize the risk of contamination, eliminate potential pathogenic microorganisms, and reduce those that can cause cheese spoilage, such as mycotoxigenic fungi. This process involves mixing one or more natural cheeses with water, emulsifying salts, and other optional ingredients, followed by heating the mixture to a temperature between 75 and 85 °C while continuously shearing [45].

A smooth and uniform melt is obtained, and the hot melt is molded into a variety of shapes and sizes appropriate to the requirement of the end user, cooled, and stored at about 8 °C [114]. For this reason, the control of temperature, time, and tests such as alkaline phosphatase for proper pasteurization is essential [115]. In order to ensure the hygienic safety of cheese, the activity of alkaline phosphatase is used throughout the world as a marker for adequate milk pasteurization [114].

### 3.9. Chemical Preservatives: Enhancing Shelf Life and Preventing Mycotoxin Growth in Cheese

Chemical preservation is the action of preventing or retarding food spoilage by changing its chemical composition, either by adding preservatives and antioxidants or promoting biochemical processes that improve preserving conditions [116]. The most commonly used chemical preservatives are sorbates, propionate, and natamycin [117]. Natamycin is used to prevent the growth of fungi in cheese-making, as noted by the study developed by Var et al. [118], in Kashar cheese, in which natamycin was used to prevent fungal growth. Furthermore, the study found that a uniform application of natamycin on cheese surfaces significantly reduced fungal contamination during storage, suggesting that combining natamycin with proper storage conditions (e.g., controlled temperature and humidity) could enhance fungal prevention. These results highlight the importance of selecting appropriate preservative concentrations and optimizing application techniques to maximize the efficacy of chemical preservatives. According to the Federal Standard of Identity of the USA, the permitted level of sorbate used in cheese production is 0.2–0.3%. This concentration is able to inhibit most fungal growth in cheese, mainly those mycotoxigenic ones [109]. Moreover, in order to control fungal growth on cheese surfaces, attempts have been made to impregnate wrapping or packaging material with fungicides or fungistatic chemicals. However, excessive use of food preservatives can cause nausea, diarrhea, and anorexia. The United States Food and Drug Administration (FDA) published a report in August 2022, which advises that the use of natamycin as an antifungal agent in cheese should not exceed 20 mg kg^−1^ in the final product [119].

### 3.10. Biological Control: Harnessing Microbial Antagonists to Combat Mycotoxins in Cheese

To extend shelf life and improve food safety, biopreservation or biocontrol has been applied. These refer to the use of natural microbiota (microorganisms that are inherently present) or controlled microbiota or their antimicrobial products in food production [120]. Due to organic acid production, competition for nutrients, and the production of other antagonistic compounds, species belonging to the *Lactococcus* and *Lactobacillus* genera are the most capable of preventing or limiting the growth of mycotoxigenic fungi on cheese [121]. Studies have been conducted on their preservative potential against fungal pathogens [122,123]. Cheong et al. [111] reported that LAB isolated from various herbs, fruits, and vegetables are able to produce antifungal agents that make these microorganisms attractive to be used as biopreservatives in cheese. Similarly, Lynch et al. [124] reported that a bacterial complement composed of eleven species of *Lactobacillus* presents antifungal activity. In the presence of this bacterial complement, cheddar cheeses exposed to natural fungi in the air benefited from the inhibition of the growth of fungi on their surfaces, also without a negative impact on the quality of the cheese. Together, these studies support the use of LAB as natural biocontrol agents to enhance cheese safety and shelf life.

### 3.11. Plant Extracts: Natural Solutions for Mycotoxin Prevention in Cheese

Natural plant extracts can provide another alternative way to protect food from the occurrence of fungi and mycotoxins due to their antioxidant, antimicrobial, and antifungal properties, which help protect food from spoilage and contamination [94]. One of the techniques that has been used in cheese-making to prevent the growth of fungi is the use of natural products. Because of their antifungal properties, herbs and essential oils have been used for the prevention of fungal growth in cheese [125].

The effect of five different concentrations of essential oil of *Ocimum gratissimum* (200, 400, 600, 800, or 1000 mg L^−1^) was assessed on cheese against six fungal species: *Aspergillus flavus*, *A. tamarii*, *F. verticillioides*, *Penicillium citrinum*, and *P. griseofulvum*. All of these fungal species were isolated from the Benin-produced Wagashi cheese, which is a soft brine-pickled cheese made from whole cow milk by adding the juice of the crushed stems of *Bryophylum*, without the use of rennet or lactic starter. It was noted that mycelia growth was reduced by increasing the essential oil level in cheese. A significant fungistatic activity against all the examined fungal species was observed with minimal inhibitory concentration values, ranging from 800 to 1000 mg L^−1^. *Penicillium citrinum*, *P. griseofulvum*, and *F. poae* species were the most susceptible fungi to the essential oil. According to the authors, the efficacy of *O. gratissimum* essential oil was attributed to its major compounds, including thymol, γ-terpinene, and p-cymene [126].

Muñoz-Tebar et al. [127] demonstrated that oregano, savory, and tarragon have significant antifungal activity against 14 fungi strains (mainly *Penicillium* and *Aspergillus*) isolated from sheep cheese. Gandomi et al. [128] evaluated the antifungal effect of *Zataria multiflora Boiss* against *Aspergillus flavus* ATCC 15546 and the production of AF in a synthetic medium such as in Iranian cheese. At 200 ppm, radial growth and sporulation were reduced by 79.4% and 92.5%, respectively. The oil also significantly suppressed mycelial growth and total AF production in broth medium for all concentrations tested (*p* < 0.05). At 150 ppm of essential oil, the mycelial growth and aflatoxin accumulation were reduced by 90% and 99.4%, respectively. However, no concentration of essential oil was able to completely inhibit the growth and total AF production in cheese. The authors suggested that the results were because of the potential substitution of the antifungal chemicals with this essential oil as a natural inhibitor of fungal growth control in food such as cheese.

Noori et al. [105] evaluated the effects of *Zataria multiflora Boiss* essential oil (EO) against growth and citrinin production by *Penicillium citrinum* ATCC 1156 were evaluated in culture media and mozzarella cheese. The growth was completely inhibited at 200 ppm on potato dextrose agar, and the minimum fungicidal concentration of the EO was estimated at 400 ppm. EO strongly suppressed the growth and citrinin formation in media in a dose-dependent manner. The authors concluded that this EO could be safely used as a preservative material on some kinds of food such as cheese to protect them from fungal contamination because it has an antifungal compound.

Mohajeri [129] investigated the impact of different concentrations of *Zateria multiflora Boiss* EO on radial growth and spore production in an agar medium. All EO concentrations showed significant inhibition of fungal growth and spore production (*p* < 0.05). At a concentration of 400 ppm, the fungal growth was completely inhibited. At concentrations of 50, 100, and 200 ppm, radial growth was reduced (52.8%, 75.3%, and 92%, respectively) and spore production was inhibited by 89.5%, 91.47%, and 100%, respectively, which seems to depend on the level of EO use. According to studies, this is possibly due to the pH of the environment, the presence of lipids that decrease the activity of hydrophobic compounds, and the presence of proteins that can lead to the binding of some compounds and the reduction of activity may affect the effectiveness of the compounds in the food system.

**Table 3:** Control strategies to prevent mycotoxin contamination.

Strategies	Advantages	Disadvantages
Sanitation of processing fabrication plant	Reduces contamination risk, improves product safety, enhances shelf life, cost-effective, builds consumer trust [33,45,110].	Labor-intensive, costly initial investment, prone to human error, limited effectiveness alone, environmental concerns [110].
Pasteurization	Destroys pathogens, reduces spoilage microorganisms, ensures consistent safety, widely accepted and regulated [114].	Energy-intensive, requires precise control, can alter flavor or texture, may not eliminate all contaminants [43].
Chemical preservatives	Effective against fungal growth, extend shelf life, easy to apply, well studied and regulated [115].	Potential health risks with excessive use, synthetic nature may deter consumers, regulated usage levels [42,112]
Biological control	Natural method, enhances food safety, sustainable, inhibits fungal growth without synthetic chemicals [128]	Requires specific microbial strains, possible flavor alteration, dependent on environmental conditions [129].
Natural plant extract	Eco-friendly, reduces fungal growth and mycotoxins, has multiple health benefits, natural alternative to chemicals [103].	Variable efficacy, dependent on concentration and type, may not fully inhibit contamination, potential sensory changes [57,102].

In summary, preventing mycotoxin contamination in cheese production requires an integrated approach involving multiple strategies. Hygiene practices play a foundational role in minimizing contamination risks, while pasteurization ensures the destruction of pathogens and spoilage microorganisms. Chemical preservatives and biological control methods effectively inhibit fungal growth and mycotoxin production, though their applications require careful regulation and monitoring. Natural solutions, including plant extracts, offer eco-friendly and health-conscious alternatives, with the potential to replace synthetic additives. By combining these preventive measures, the cheese production process can achieve enhanced safety, extended shelf life, and improved consumer confidence, addressing both industry and public health concerns.

## 4. Conclusions

Despite the global importance of cheese production, it is highly susceptible to contamination by mycotoxin-producing fungi. The most common stable mycotoxins in cheese are AFM_1_, CIT, CPA, ROQ-C, and MPA. However, there is limited evidence regarding the factors that determine the growth of mycotoxigenic fungi, making it critical to develop strategies to mitigate the growth of fungi capable of producing mycotoxins. To address this issue globally, it is fundamental to answer key questions such as (I) Which compounds present in cheese induce or inhibit the gene expression of mycotoxins? (II) What physicochemical factors influence the production of mycotoxins?

Toxicological investigations are necessary to develop new tools to understand the mechanisms of toxicity for these mycotoxins. Additionally, further knowledge of mycotoxin biosynthetic pathways could aid in developing innovative analytical methodologies to elucidate the physicochemical factors involved and to control mycotoxin production in cheese. Moreover, more research is needed on masked mycotoxins, along with the advancement of sensitive, specific, rapid, low-cost, and portable detection methods, such as biosensors, for application in the cheese industry.

## Figures and Tables

**Table 1:** Worldwide occurrence of fungi on cheese.

Type of Cheese	Origin	Country	Fungal Strains	Reference
Handmade, semi-hard, and mature	Storehouse	Spain	*Alternaria*, *Aspergillus*, *Fusarium*, *Geotrichum*, *Penicillium* sp.	[23]
Blue	Supermarket	Italy	*Penicillium glabrum*, *P. roqueforti*, and *P. cyclopium*	[51]
Goat, Saint Marcellin, and Soigno	Supermarket	France	*Penicillium citrinum* and *P. expansum*	[52]
Blue moldy Tulum	Supermarket	Turkey	*Penicillium* *roqueforti*	[24]
Nalžovy	Artisan	Czech Republic	*Penicillium nalgiovens*	[53]
Camembert cheese-ripening	Storage	Canada	*Penicillium camemberti* and *G. candidum*	[54]
Handmade and semi-hard	Supermarket	Italy	*Aspergillus versicolor*, *A. niger*, *P. camemberti*, *P. citrinum*, *P. crustosum*, *P. nalgiovense*, *P. nordicum* and *P. roqueforti.*	[41]
Hard	Supermarket	Serbia	*Aspergillus flavus*, *A. parasiticus*	[5]
Civil	Market	Turkey	*Penicillium* *roqueforti*	[55]
Blue-veined cheese	Supermarket	Denmark, France, Italy, Ireland, Netherlands and Scotland	*Penicillium roqueforti*	[51]
Ras cheese (romy)	Market	Egypt	*Aspergillus ochraceus*, *A. alliaceus*, *A. oryzae*, *A. niger*, *A. nidulans*, *A. flavus*, *A. glaucus*, *A. flavipes*, and *Penicillium* sp.	[56]
Grana cheese	Storehouse	Italy	*Aspergillus versicolor*, *P. camemberti*, *P. citrinum*, *P. crustosum*, *P. nalgiovense*, *P. nordicum*, *P. roqueforti*, and *P. verrucosum*	[25]
Cave cheese	Storehouse	Italy	*Penicillium* *gravinicasei*	[28]
Grana cheese	Storehouse	Italy	*Aspergillus flavus*, *P. crustosum*, *P. verrucosum*, and *P. solitum*	[57]

**Table 2:** Mycotoxins reported in cheese.

Type of Cheese	Mycotoxin	Country	References
Blue cheese	ROQ-C; MPA; PAT, OTA; and PR	Denmark, France, Italy, Ireland, Netherlands, Turkey, and Scotland, USA	[9,22,27,64,65,66,67]
Semi-hard	OTA; PAT, AFM_1_	Italy, Serbia	[41,68]
Goat, Saint Marcellin, Soigno	CIT	France	[69]
Civil	ROQ-C; MPA; PAT	Turkey	[68]
Traditional	AFM_1_	Iran	[70]
Ras	STC	Egypt	[30,36,59]
Cave	OTA, CIT, PAT, STC or AFB1	Italy	[30,38,59]
Grana	OTA; ROQ-C; PR toxin; PA; CIT	Italy	[25,56,57]
Camembert	CPA; AFM_1_	Canada, Lebanon	[2,30,52,71]
Cheese	AFM_1_; CPA	Brazil, Lebanon	[9,72]
Cheese	Enniatin A/B	Slovakia, Nigeria	[73,74]

Afs (Aflatoxins), CIT (Citrinin), OTA (Ochratoxin A), PAT (Patulin), ROQ-C (Roquefortine C) and STC (sterigmatocystin), and CPA (Cyclopiazonic Acid).

## Data Availability

No new data were created or analyzed in this study. Data sharing is not applicable to this article.
